# Dating Aggression and Observed Behaviors in a Nonconflictual Situation: The Role of Negative Anticipation

**DOI:** 10.1177/08862605211035877

**Published:** 2021-08-03

**Authors:** Marie-Ève Daspe, Reout Arbel, Hannah F. Rasmussen, Gayla Margolin

**Affiliations:** 1 Université de Montréal, Québec, Canada; 2 University of Haïfa, Israel; 3 University of Southern California, Los Angeles, CA, USA

**Keywords:** dating aggression, dating couples, couple interactions, observed behaviors, anticipation

## Abstract

Past observational studies highlight meaningful behavioral differences between aggressive and nonaggressive couples during conflict interactions. However, research is needed on how aggressive couples communicate in other, nonconflictual interactional contexts. This study investigates how dating partners’ perpetration of physical aggression relates to observed behaviors during a laboratory-based discussion during which dating couples planned a date together. We also investigated whether negative anticipation of the upcoming discussion influences dating partners’ observed behaviors. Results showed that perpetration of dating aggression from one partner is linked to more negative behaviors from the other partner during the discussion. This association, however, is moderated by negative anticipation of the discussion; the link between aggression from one’s partner and negative behaviors is significant at high levels (+1 *SD*) but not at low levels (–1 *SD*)of negative anticipation. One’s own dating aggression also relates to fewer positive behaviors during the discussion. Findings suggest that couple aggression spills over to and potentially degrades the discussion of even nonthreatening, potentially enjoyable communications. Results also underscore negative anticipation of an interaction as a potential risky process that increases the likelihood of antagonistic exchanges between partners. The discussion addresses putative pathways between partner aggression and generalized communication patterns, and potential bi-directional effects with negative anticipation. We also discuss practical implications and targets of intervention to counteract the establishment of problematic communication dynamics in young couples.

Aggression within romantic relationships is a complex and serious issue. Young adults are particularly at risk of perpetrating dating aggression, as prevalence estimates peak in the early twenties (Johnson et al., 2015): between 17% and 48% of young women and 10% and 39% of young men report having been physically aggressive toward a dating partner (see Dardis et al., 2015, for a review). In addition to a host of negative consequences for victims’ psychological and mental health (Exner-Cortens et al., 2013), aggressive incidents can evolve into chronic patterns of aggression that persist over time and across developmental stages (Gómez, 2011; Lorber & O’leary, 2004).

Direct observation of couple interactions shows that aggressive couples exhibit more hostile behaviors, have more trouble exiting reciprocal negative exchanges, and show fewer positive behaviors than nonaggressive couples when discussing areas of conflicts in their relationship (Burman et al., 1993; Friend et al., 2017; Jacobson et al., 1994). Although observational research sheds light on the problematic ways aggressive couples manage disagreements, the reliance on conflict discussions paradigms provides little information about how the perpetration of aggression is linked to behaviors in other types of partner interactions. Given that couple relationships do not consist of solely conflictual or negatively-charged discussions, it is necessary to study the impact of aggression in other interactional contexts (Holtzworth-Munroe et al., 1997). Relatedly, as adverse communication patterns during conflict discussions are potential behavioral warning signs of aggression within romantic relationships, it is interesting to investigate whether these warning signs are also present in other types of couple interactions. Examining aggressive partners’ behaviors during nonconflictual interactions that do not inherently call for hostile behaviors could help detect couples dealing with violent dynamics through a wider range of interactional contexts.

In the current study, we examined how perpetration of physical dating aggression relates to observed behaviors during a laboratory-based discussion in which young-adult couples plan a date together. In addition, as partners’ expectations about upcoming couple interactions have been shown to influence the behaviors they exhibit (Sanford, 2006), we also examined whether negative anticipation prior to the discussion has an impact on the strength of the associations between perpetration of physical aggression and observed behaviors. This investigation is important given that expectations about upcoming interactions constitute proximal factors that potentially sustain more negative exchanges between partners.

## Aggression Within Dating Relationships

Correlates of dating aggression perpetration encompass various dispositional variables: personality characteristics (e.g., low empathy), history of experiencing child abuse, witnessing parental aggression, attitudes towards aggression, and so forth (See Dardis et al., 2015, for a review). Yet, given its inherently interpersonal nature, partner aggression must be considered within the relational context in which it takes place (Bartholomew & Cobb, 2011). Research has found that aggression is more stable within than across relationships, suggesting that a dyadic perspective on partner aggression is warranted (Capaldi et al., 2003). Moreover, reciprocal aggression between partners is more common in dating relationships than unilateral aggression where one partner is the perpetrator and the other is the victim (Daff et al., 2018; Langhinrichsen-Rohling et al., 2012). In heterosexual couples, both women and men are reported to display aggressive behaviors toward their partner, although women are more likely to suffer injuries (Archer, 2000) and a host of negative consequences from victimization (Caldwell et al., 2012). One underlying factor of reciprocal aggression is the fact that having experienced physical aggression from an identified dating partner is a strong predictor of perpetration of physical aggression toward that partner (O'Leary & Slep, 2003). Dyadic perspectives on partner aggression (Bartholomew & Cobb, 2011), therefore, seek to understand couple dynamics associated with aggression within romantic relationships. From this point of view, observation of couple interactions is likely to provide the most direct evidence of dyadic processes distinguishing aggressive and nonaggressive couples (Bartholomew et al., 2015).

## Behavioral Patterns in Aggressive Couples

Examination of the way couples attempt to resolve conflict has highlighted meaningful behavioral differences between aggressive and nonaggressive couples. Direct observation of couple interactions suggest that aggressive couples display poorer communications, characterized by hostility, anger, contempt, belligerence, and low problem-description, and fewer positive behaviors such as warmth, validation, and affection (Friend et al., 2017; Gordis et al., 2005; Jacobson et al., 1994). The demand-withdraw behavioral pattern, in which one partner actively pressurizes the other through requests and criticism while the latter defensively retreats in passive inaction, has also been observed in violent couples (Berns et al., 1999a,b). Moreover, aggressive couples tend to engage in negative reciprocity: partners are more likely to adopt a hostile stance in response to each other’s displays of hostility. Aggressive, compared to nonaggressive, couples also take longer to exit negative reciprocal exchanges (Burman et al., 1993; Cordova et al., 1993).

Observational research has traditionally focused on married couples with fewer studies investigating communication behaviors in young dating couples. One exception to this is the work of Capaldi and Crosby (1997) and Capaldi et al. (2007) who examined observed behaviors during various interaction tasks in at-risk dating couples where the male partner was recruited from a high school in a high-delinquency neighborhood. They found instances of hostile physical contacts (e.g., poking, shoving) from both partners during couple interactions. Moreover, hostile physical contacts were significantly associated with higher self-reported physical aggression in the relationship and with a higher prevalence of injuries resulting from violent episodes when partners were physically hostile with one another (Capaldi et al., 2007). More recently, Paradis et al. (2015) examined problem-solving communication behaviors in a sample of 39 dating couples aged 15 to 20. Accounting for both young men and women’s dating aggression perpetration in the relationship, they found that behaviors displayed while discussing a disagreement were related to the partner’s aggression but not to the individual’s own aggression. Specifically, women and men’s display of negative behaviors—a composite score of conflict, withdrawal, and negative affect—were positively associated with their partner’s dating aggression perpetration, whereas women’s positive behaviors—a composite score of communication skills, validation, problem-solving behaviors, and positive affect—were negatively associated with their partner’s dating aggression perpetration. These partner effects demonstrate how both partners’ aggression in the relationship relates to the quality of their behavioral exchanges and underscores the need to use a dyadic framework for studying dating aggression. These findings also suggest that the problematic behavioral patterns observed in married couples also deserve attention in younger dating couples. Although Paradis et al. (2015) were, to our knowledge, the first to examine observed behaviors in dating couples from a dyadic perspective, their study relied on a small sample of couples and leaves unanswered questions regarding links between perpetration of aggression in the relationship and behaviors surrounding nonconflictual topics. The present study expands on these previous findings by examining associations between perpetration of aggression and observed behaviors during a nonconflictual couple interaction, using a larger sample of young adult dating couples.

The bulk of evidence concerning behavioral patterns in aggressive couples comes from observation of partners discussing areas of disagreement. Although the conflict discussion paradigm is relevant for understanding the type of conflict-management style that is more likely to culminate in aggression, it does not reveal how aggressive couples negotiate other areas of their relationship. This is important given that aggressive partners may show pervasive dysfunctional communication patterns that are not restricted to conflict situations. For instance, in a comparison of aggressive and nonaggressive husbands instructed to provide social support while their wife discussed a personal problem (e.g., career, friendships, personal habits, etc.), the aggressive husbands were less positive and more domineering, contemptuous, and upset (Holtzworth-Munroe et al., 1997). They also displayed more anger and tension, and were more critical of their wives’ problems and suggestions of possible solutions than nonaggressive, nondistressed husbands. With the goal of investigating communication behaviors in aggressive and nonaggressive couples beyond conflict situations, the current study focuses on a neutral interactional context: a discussion to plan a date. We aim to examine whether nonconflictual and seemingly nonthreatening interactions can nevertheless be challenging for couples dealing with aggression.

## The Role of Anticipation

In terms of behaviors displayed during a specific exchange, history of aggression within the relationship might be considered a somewhat distal contextual factor. In this view, more proximal factors are likely to be especially meaningful in shaping how partners behave in the here-and-now of an interaction. According to Bradbury and Fincham’s (1991) contextual model of marital interactions, expectations about an upcoming interaction is one proximal factor likely to influence the type of behaviors partners will display. In support of this assumption, changes in cognitive appraisals of couple interactions are, in fact, associated with changes in behaviors from one conflict discussion to the next (Sanford, 2006). It is, therefore, argued that couple interactions must be understood in light of the appraisals and expectations that each partner brings to these interactions (Fincham et al., 1995). Finding that greater physiological arousal prior to marital interactions predicted decline in marital satisfaction over 3 years, Levenson and Gottman (1985) attributed the higher physiological arousal to negative appraisals of the upcoming discussions. Negative anticipation of an upcoming interaction might, therefore, adversely impact the quality of couple interactions by prompting more antagonistic exchanges between partners. One objective of the present study is thus to examine whether anticipation about the upcoming discussion moderates the association between prior aggression and couples’ behavioral patterns during a neutral interaction.

## Present Study

The goal of the current study is to investigate whether partners’ perpetration of dating aggression is related to their behaviors during a nonconflictual couple interaction. We objectively assessed both partners’ negative and positive behaviors during a laboratory-based discussion in which they were asked to plan a date together. We also examined whether negative anticipation, reported immediately prior to the discussion, moderates the associations between dating aggression perpetration and observed behaviors. Using actor-partner interdependence models (APIMs), we investigated both actor effects (i.e., the association between one’s own dating aggression perpetration and own behaviors) and partner effects (i.e., the association between the partner’s dating aggression perpetration and own behaviors). Based on previous literature showing more adversarial interactions among aggressive couples compared to nonaggressive couples (Burman et al., 1993; Cordova et al., 1993; Friend et al., 2017; Gordis et al., 2005; Jacobson et al., 1994), we first hypothesized significant actor and partner effects: one’s own and one’s partner’s physical aggression would be associated with more negative behaviors and fewer positive behaviors (HO1) during the date planning discussion. Second, in light of previous studies highlighting the influence of expectations about an upcoming interaction in shaping behaviors (Fincham et al., 1995; Sanford, 2006), we hypothesized that the associations between aggression and observed behaviors would be moderated by negative anticipation of the discussion (HO2): specifically, for actor effects, the association between one’s own physical aggression and behaviors during the discussion would be stronger at high, compared to low, levels of own negative anticipation. Similarly, for partner effects, the cross-partner association between the partner’s physical aggression and own behaviors during the discussion would be stronger at high levels of own negative anticipation. Although we expected the hypotheses to apply to both women and men, we examined the potential influence of sex on the findings.

## Method

### Overview

The current study is part of larger laboratory-based procedure studying young adults’ relationship functioning. Dating couples were invited to the lab to engage in a series of interactional tasks lasting 4-5 hours for which they were compensated $125. The first task was the date planning discussion, which is the focus here. The study was approved by the Institutional Review Board of the University of Southern California.

### Participants

Of the 117 heterosexual dating couples participating in the larger study, one couple was excluded due to missing pre-discussion appraisal data, resulting in an analytic sample of 116 couples (232 participants). Participants on average were 22.59 years old (*SD* = 2.42). Mean length of the relationship was 29.78 months (*SD* = 23.70) and 43.1% of participants were living together. The sample was ethnically diverse with 15.1% identifying as African-American/Black, 25% Hispanic, 27.6% Caucasian, 12.5% Asian, 15.9% multi-racial, and 3.9% other. The majority of couples (*n* = 87) were recruited through flyers and online notices. To be eligible, couples needed to be together for at least two months, and between ages 18 and 25 inclusive. The remaining couples were recruited from a follow-up to a longitudinal study on family functioning and adolescent development and, again, were eligible here if they had a dating partner of two months or longer who agreed to participate. Participants from the longitudinal sample did not differ from newly recruited participants on age or length of the relationship but were less likely to live with their partner, χ^2^(1) = 10.55, *p* = .001.

### Procedure

For the 5-minute date planning discussion, participants were given the following instruction: “We would like you to plan a special date together. Please think about what would be a fun date for the two of you—assume that you are doing this on an evening when you don’t have school or work the next day. Share your ideas with one another about what this date would look like.” Immediately prior to the discussion, each partner completed a survey assessing anticipation of the upcoming discussion. Later in the lab procedures, participants individually completed a number of questionnaires, including a measure of dating aggression.

### Measures

#### Observed behaviors.

Partners’ displays of positive and negative behaviors during the date planning discussion were assessed through a coding system developed for the current study. The four negative behaviors included criticism (of other person or other person’s ideas), irritation (in voice, facial expression, or vocal content), self-focused direction (promoting one’s own ideas without trying to build on the partner’s input), and withdrawal (pulling back verbally or nonverbally from discussion). The three positive behaviors included collaboration (seeking the partner’s input and negotiation in an inclusive way), excitement (in tone and content), and praise (of the partner or the partner’s ideas, reflecting positive feeling about the relationship). A subset of videotaped discussions was selected to pilot the coding system and train coders. Research assistants watched videos individually and met as a group with the first author in order to clarify, remove, or modify codes that were unclear, and add relevant examples of behaviors to refine the coding scheme. After the piloting and training period, two coders independently watched the video-recordings once (or twice, when needed) and separately rated each partner. The coding team met weekly to discuss coding questions in order to avoid coding drift. Behaviors were coded on a 4-point scale ranging from 0 (*not at all*) to 3 (*a lot*). The four negative behaviors and the three positive behaviors were first averaged within each coder for composite negative and positive scores, and then averaged across coders. Interrater reliabilities calculated through intraclass correlation coefficients were .71 for negative behaviors and .73 for positive behaviors, respectively. Overall, 75.0% of women and 76.1% of men displayed instances of negative behaviors (i.e., composite score above 0), and 99.1% of women and 99.1% of men displayed instances of positive behaviors during the date planning discussion. No significant sex differences emerged for negative or positive scores.

#### Negative anticipation.

To assess pre-discussion appraisals of the date planning discussion, we used a modified version of a cognitive appraisal measure (Berry Mendes et al., 2007) that included six items: “I’m looking forward to this discussion”, “I’m dreading this discussion”, “I think I’ll do a good job of getting my points across in this discussion”, “I may have a hard time saying what I want to say in this discussion”, “Something good is likely to come out of this discussion”, and “I doubt this discussion will be useful”. Participants rated each item on a 7-point scale ranging from 1 (*disagree strongly*) to 7 (*agree strongly*) with the three positive items reversed coded and all items averaged to obtain a score of negative anticipation (Cronbach’s alpha = .75).

#### Dating aggression.

Physical dating aggression ever experienced within the current relationship was assessed using the 9 physical aggression items (e.g., “Pushed, shoved or shook your partner”) from the 65-item *How Partners and Friends Treat Each Other* (Bennett et al., 2011), which had been adapted in part from the *Conflict in Adolescent Dating Relationship Inventory* (Wolfe et al., 2001) and the *Domestic Conflict Inventory* (Margolin et al., 1998). Participants went through the scale twice, first reporting their own aggression perpetration and then reporting the partner’s aggression perpetration to them. For purposes here, we categorized each partner as either 0 (no physical aggression perpetration) or 1 (at least one occurrence of any physically aggressive behaviors) based on either reporter’s endorsement of any physical aggression perpetration items; 25.9% of women and 13.8% of men perpetrated physical aggression toward their partner.

### Analytic Plan

APIMs (Kenny et al., 2006; conducted in SPSS 20) were used to take account of the nonindependence between partners and to simultaneously examine actor effects (i.e., the association between own dating aggression perpetration and own behaviors) and partner effects (i.e., the association between partner’s dating aggression perpetration and own behaviors) in one comprehensive model (HO1). We used multilevel modeling to nest individuals (level 1 actor dating aggression and level 1 partner dating aggression) within couples (level 2). Negative and positive behaviors were examined in separate models. Because opposite-sex dyads are theoretically distinguishable, we included sex (–1 = Women; 1 = Men) in every analysis to examine sex differences. Sex did not moderate any associations for HO1, so we present those results across women and men.

To investigate whether negative anticipation of the discussion moderated the actor and partner effects of dating aggression perpetration on behaviors (HO2), we conducted actor-partner moderation models (Garcia et al., 2015) that test the interaction between actor negative anticipation and dating aggression (both actor and partner); as expectations are inherently intra-individual processes, we did not expect a moderation effect for partner negative anticipation and thus focused exclusively on the actor negative anticipation (Level 1). In the same model, we tested the interaction between actor negative anticipation with: (a) actor dating aggression perpetration; and (b) partner dating aggression perpetration. Negative anticipation of the discussion was grand mean centered. Simple slope tests for high (+1 *SD*) and low (–1 *SD*) levels of negative anticipation were conducted for significant interactions. We included sex (–1 = Women; 1 = Men) as a moderator of the main effects and of the two-way interactions between dating aggression and negative anticipation. Sex did not moderate any associations for HO2, so we present those results across women and men. All analyses adjusted for length of the relationship (Level 2) and cohabitation (Level 2).

## Results

### Descriptive Statistics and Correlations

Descriptive statistics as well as correlations between the study variables are presented in Table 1. Rates of physical aggression in the current sample are consistent with those found in previous studies (see Dardis et al., 2015, for a review). The significant cross-partner correlation for physical aggression perpetration suggests largely reciprocal violence. Cross-partner correlations were also significant for negative behaviors, positive behaviors, and negative anticipation. Women’s physical aggression perpetration was positively correlated with men’s negative behaviors, but unrelated to other variables. Men’s physical aggression perpetration was negatively correlated with men’s positive behaviors, but unrelated to other variables. Negative and positive behaviors during the date planning discussion were inversely correlated with each other, both within and across partners. Finally, men’s positive behaviors were negatively associated with men’s negative anticipation of the discussion. Tests of sex differences on the study variables revealed that a greater proportion of women than men perpetrated physical aggression, χ^2^(1) = 5.32, *p* = .032. Men reported higher levels of negative anticipation than women, *t*(115) = –2.52, *p* = .013. No sex differences emerged regarding negative and positive behaviors during the date planning discussion.

**Table 1.** Correlations and Descriptive Statistics for All Study Variables.

	1	2	3	4	5	6	7	8	9	10
1. W physical aggression^a^	-									
2. W negative behaviors^b^	.02	-								
3. W positive behaviors^b^	–.08	–.48^***^	-							
4. W negative anticipation	.07	.18	–.13	-						
5. M physical aggression^a^	.34^***^	.09	–.16	.15	-					
6. M negative behaviors^b^	.24^*^	.40^***^	–.31^**^	.07	.12	-				
7. M positive behaviors^b^	–.10	–.31^**^	.65^***^	–-.12	–.22^*^	–.48^***^	-			
8. M negative anticipation	.01	.11	–.17	.20^*^	.16	.18	–.24^*^	-		
9. Relationship length^c^	–.05	.09	-.11	–.04	.04	.10	–.17	.11	-	
10. Cohabiting^a^	.12	–.07	–.02	–.02	.01	–.13	.04	.08	.09	-
	*%* (n)	*M* (SD)	*M* (SD)	*M* (SD)	*%* (n)	*M* (SD)	*M* (SD)	*M* (SD)	*M* (SD)	% (*n*)
	25.9 (30)	.36 (.39)	1.20 (.45)	1.89 (.80)	13.80 (16)	.31 (.31)	1.14 (.41)	2.13 (.86)	29.78 (23.70)	43.10 (50)

*Notes.* * *p* < .05. ** *p* < .01. *** *p* < .001. W = Women; M = Men. ^a^Point-biserial correlations. ^b^Coded behaviors during the date planning discussion. ^c^Relationship length is assessed in months.

### Main Effect of Aggression Perpetration on Behaviors During the Date Planning Discussion

We first examined associations between physical aggression perpetration, both actor’s and partner’s, and behaviors during the date planning discussion (HO1; see Table 2, Model 1). For negative behaviors, no significant actor effect of physical aggression perpetration emerged. However, we observed a significant partner effect, indicating that being on the receiving end of physical aggression from one’s partner is linked to more negative behaviors during the discussion. The significant main effect of sex indicated that men displayed less negative behaviors than women, but sex did not moderate any of the findings. For positive behaviors, results revealed a significant actor effect, indicating that one’s own physical aggression is related to fewer positive behaviors during the date planning discussion. However, no significant partner effect was found. Sex did not show a main or a moderating effect, indicating that the inverse association between physical aggression perpetration and positive communication behaviors did not differ across women and men.

**Table 2.** Actor-Partner Interdependence Moderation Models for Negative and Positive Behaviors During the Date Planning Discussion.

Predictors	Model 1 HO1: Main Effect of Aggression			Model 2 HO2: Moderating Effect of Anticipation		
	*b*	*SE*	*p*	*b*	*SE*	*p*
Negative behaviors						
Sex	–**.04**	**.02**	**.044**	–**.05**	**.02**	**.023**
A aggression	.02	.06	.727	-.06	.06	.293
P aggression	**.14**	**.06**	**.029**	**.13**	**.06**	**.040**
A aggression * Sex	.01	.07	.889	–.02	.07	.817
P aggression * Sex	.04	.07	.546	.04	.07	.574
A anticipation				.03	.03	.406
A anticipation * Sex				–.01	.03	.632
A aggression * A anticipation				–.03	.08	.732
P aggression * A anticipation				**.25**	**.07**	**.001**
A aggression * A anticipation * Sex				.13	.08	.116
P aggression * A anticipation * Sex				–.11	.09	.179
Length of the relationship	.00	.00	.134	.00	.00	.107
Cohabitation	–.10	.05	.071	–.11	.05	.042
Positive behaviors						
Sex	-.02	.02	.232	–.02	.02	.342
A aggression	–**.14**	**.07**	**.041**	–.10	.07	.161
P aggression	–.11	.07	.114	–.10	.07	.166
A aggression * Sex	–.09	.08	.259	–.06	.09	.498
P aggression * Sex	.07	.09	.431	.06	.09	.494
A anticipation				–.04	.03	.280
A anticipation * Sex				–.01	.03	.699
A aggression * A anticipation				–.08	.08	.315
P aggression * A anticipation				.00	.08	.997
A aggression * A anticipation * Sex				–.07	.09	.480
P aggression * A anticipation * Sex				.04	.09	.681
Length of the relationship	–.00	.00	.090	–.00	.00	.075
Cohabitation	.03	.07	.665	.05	.07	.525

*Notes*. A = Actor; P = Partner; Aggression = Physical Aggression Perpetration; Anticipation = Negative Anticipation. Sex is effect coded (Women = –1 and Men = 1) Results in bold are significant at p < .05.

### The Moderating Role of Negative Anticipation of the Discussion

We next investigated whether actors’ negative anticipation of the discussion influenced the strength of the associations between physical aggression perpetration and behaviors (HO2; see Table 2, Model 2). For negative behaviors, a significant interaction emerged between partners’ physical aggression and actor negative anticipation.

**Figure 1.**
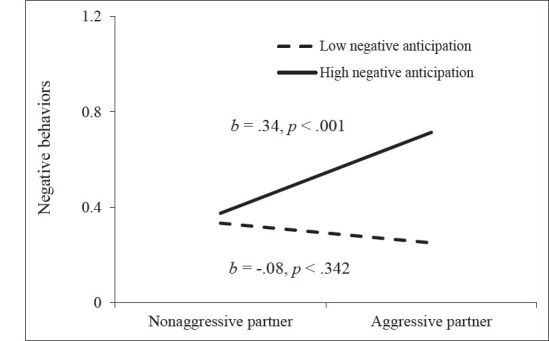
Moderating effect of actor’s negative anticipation in the association between partner aggression perpetration and negative behaviors across women and men.

To decompose the significant partner aggression X actor negative anticipation interaction, we tested simple slopes of the association between partners’ physical aggression perpetration and negative behaviors at high (+1 SD) and low (–1 SD) levels of actor negative anticipation. As illustrated in Figure 1, partners’ physical aggression perpetration predicted more negative behaviors only for participants who showed high negative anticipation of the discussion. For participants who showed low negative anticipation, partner aggression perpetration was unrelated to their negative behaviors during the discussion. A main effect of sex was still observed; however, sex did not moderate any of the findings.

For positive behaviors, negative anticipation showed no main or moderating effect. Sex did not emerge as a significant moderator.

## Discussion

Although prior studies demonstrate that relationship aggression plays a role in conversations about contentious issues, much less is known about its links with communication behaviors during other types of couple interactions. Our findings suggest that the link between aggression and communication is not restricted to conflict: for aggressive couples, seemingly nonthreatening discussions, such as planning a date, also elicit more negative behaviors and less positive behaviors. Dating aggression thus appears to permeate wide-ranging topics of discussion and potentially increases risks for more adversarial exchanges even in nonconflictual interactional contexts. In partial support for our hypotheses (HO1), we found that a history of physical aggression from one partner was linked to the other partner’s criticism, irritation, self-focus, and withdrawal during a discussion about an ideal date together. Expectations about the upcoming discussion further moderated this effect (HO2). Specifically, one’s partner physical aggression perpetration was associated with negative behaviors only when apprehensions about the discussion were high. Our data also indicate that a person’s own aggression was not related to their negative behaviors during this discussion. However, one’s own physical aggression perpetration within the relationship related to less collaboration, excitement and praise during the date planning discussion. These links did not vary as a function of negative anticipation of the discussion and one partner’s perpetration of physical aggression did not relate to the other partner’s positive behaviors.

Even though it is well-known that aggression has a different effect on women and men, with women being more likely to suffer negative consequences (see Caldwell et al., 2012, for a review), sex did not play a consistent role in our study. Based on coded behaviors, men displayed less negative behaviors than women during the date planning discussion; however, sex did not influence any of the associations between aggression, negative anticipation, and observed behaviors.

### Direct Effects of Aggression

The findings here extend prior data supporting the importance of using a dyadic framework to examine physical aggression in romantic relationships (Bartholomew & Cobb, 2011). First, our partner effect linking physical aggression to negative behaviors is consistent with findings on dating couples from Paradis et al. (2015) who also showed that: (a) aggression from the partner was associated with negative behaviors during dating couple interactions and (b) one’s own perpetration of relationship aggression was not associated with negative communication behaviors. Beyond dating couples, the cross-partner effects also are in line with past observational research showing that wives of violent husbands display anger and hostility during conflictual interactions (Burman et al., 1993; Cordova et al., 1993; Jacobson et al., 1994); in those studies, however, wives’ own violence was not taken into account. These cross-partner effects highlight the meaningfulness of capturing dyadic as opposed to individual processes when understanding the long reach of relationship aggression.

For positive behaviors, we found only actor, and not partner, effects. These results contrast with Paradis et al. (2015) who reported only cross-partner effects of partner aggression, but not own aggression, linked to less positive behaviors during a conflict discussion. This discrepancy might be due to the different types of interactions in the two studies (conflictual vs. nonconflictual). The current study examined a set of positive behaviors that were particularly meaningful to the date planning discussion: collaboration, excitement, and praise of the partner, partner’s idea, or relationship. These positive behaviors are only partially overlapping with those typically exhibited and coded in studies with conflict discussions (Burman et al., 1993; Cordova et al., 1993), such as humor, affection, validation, approbation, compromise, etc. In addition, the meaning and manifestation of these behaviors are likely to vary depending on whether they are displayed during conflictual or nonconflictual situations (e.g., compromising regarding a highly contentious issues versus a pleasant activity to do together).

The actor effect for the link between aggression and fewer positive behaviors observed in the context of the date planning discussion could be explained by control and power dynamics in aggressive relationships (Hamberger et al., 2017; Johnson, 2006), which are antithetical to the positive alliance represented in our positive codes. Despite the fact that the date planning discussion is not inherently a problem-solving task, it still requires some degree of negotiation from partners to discuss and eventually agree with a shared plan. Although most couples seemed to enjoy exchanging ideas about pleasant activities to do together, others veered into contentious issues such as differences in how they spend money, lack of common interests, or dissatisfaction with time spent together. It this context, it might be challenging for aggressive partners to adopt a collaborative posture of openness and interest towards the other’s input and preferences.

### Negative Anticipation of the Upcoming Discussion

In partial support of our second hypothesis, findings show the importance of expectations about an upcoming interaction as they seemed to prompt negative behaviors. The association between partner aggression perpetration and negative behaviors was observed only at high levels of negative anticipation, suggesting that proximal influences ultimately shape the quality of partners behavioral exchanges. This is consistent with the contextual model of marital interactions (Bradbury & Fincham, 1991), which posits that appraisal of an upcoming interaction influences partner behaviors. Here, having negative expectations when anticipating planning a date with an aggressive partner seemed to predispose the person to behave in a more critical, irritated, self-focused, and withdrawing fashion. These findings shed light on a potential risky process that may perpetuate aggression within dating couples by increasing partners’ likelihood to engage in harmful exchanges. It can potentially lead to the establishment of a more antagonistic interactional style within the relationship, that in turn increases further the risk for dating aggression.

It is worth noting, however, that one’s own or the partner’s physical aggression was not associated with negative anticipation. This raises the question of how these expectations develop. One assumption is that couples who have repeatedly experienced aversive exchanges establish pervasive negative expectations about their interactions in general. Although the current study does not allow us to directly examine this possibility, it can be argued that the behaviors observed during the laboratory discussion are a valid sample of how partners communicate when engaging in similar discussions in real life. Our data, however, only suggest a negative correlation between negative anticipation of the discussion and positive behaviors in men. Future studies should directly examine, along with other potential precursors, the influence of past behavioral exchanges on the development of expectations about future interactions. This is important, from a clinical point of view, to understand, and intervene on, the larger relational context that predispose partners to negatively apprehend these types of interactions. Knowing where these negative expectations come from (i.e., legitimate caution given previous adverse experiences in the relationship) seems necessary to tackle them in a safe and appropriate way. Concurrently with the typical goal of improving communication skills, this could help counteract cycles of antagonistic behaviors and foster constructive communication, through which a healthier bonding can emerge.

### Limitations and Implications of the Study

The findings of the current study must be interpreted in light of several limitations. First, the cross-sectional design precludes conclusions about causality as well as direction of the associations examined. Future studies could test how anticipation of upcoming interactions and observed behaviors relate to future dating aggression perpetration. Second, because the distribution of the physical dating aggression variable was highly skewed, scores were dichotomized. Therefore, one instance of aggressive behavior cannot be distinguished from repeated dating aggression. In addition, these scores were derived based on either reporter’s endorsement of any physical aggression perpetration item. Although this method prevents biases regarding underreporting of aggression, it results in a relatively un-nuanced assessment of physical dating aggression in the current study. Third, and in a parallel way, the combining of positive (e.g., collaboration and excitement) and negative codes (e.g., criticism and withdrawal) might blur different types of behavioral responses to dating aggression. Fourth, partners were asked to plan an ideal date together. This might limit the generalizability of our data to discussions about a real date, to which time and financial constraints are inherent. We nevertheless elected for this specific task in order to allow partners to be imaginative and fanciful, and to engage in an enjoyable interaction. Finally, regarding diversity, although participants in this study were ethnically diverse, our sample size did not allow for specific comparisons of our findings across ethnic groups. In addition, we could not be inclusive of sexual orientations. As only four same-sex dyads participated in the larger study, we were unable to examine our research question on this specific subgroup. Future research should focus recruitment efforts on same-sex dyads as they are largely underrepresented in couple research, and especially in observational research.

Despite these limitations, our study highlights the importance of investigating the link between dating aggression and observed couple interactions across different contexts and behaviors. Although the conflict discussion paradigm is dominant in observational research, other types of situations that couples commonly encounter, such as neutral or positive interactions, situations soliciting partner support (Holtzworth-Munroe et al., 1997), and decision-making tasks, also deserve attention. The focus on conflict discussions also is found in clinical applications. Interventions for aggressive couples mainly focus on building nonviolent conflict-resolution skills (Bradley et al., 2014; Heyman & Neidig, 1997; Stith et al., 2012). Although unarguably an essential component, couple treatments for aggressive couples might benefit from dealing with a wider range of interactions, beyond conflictual ones. The current study’s focus on young-adult couples is also relevant to understand interactional patterns associated with aggression earlier in individuals’ relational development and perhaps preventing the establishment of enduring violent dynamics across adulthood. Intervening on a wide range of topics, and accounting for partners’ anticipation of their interactions appears important to help dating couples develop alternative and healthier ways of relating.

## Author Biographies

**Marie-Ève Daspe**, PhD, is an assistant professor in the Department of Psychology at the Université de Montréal. Her work focuses on psychophysiological correlates of intimate partner violence. She also studies the impacts of digital technologies on relationship functioning among adolescents and young adults.

**Reout Arbel**, PhD, is an assistant professor in the Department of Counseling and Human Development at the University of Haifa. Her research examines how social connections shape individual responsivity to stress across the lifespan. She seeks to unravel trajectories of risk and resilience using diverse methodologies that facilitate uncovering the social regulation of stress across contexts.

**Hannah F. Rasmussen**, MA, is a doctoral candidate in the Department of Psychology at the University of Southern California. Her research aims to provide new knowledge about physiological, behavioral, and emotional characteristics that create risk for—or, more importantly, that prevent—aggression in parent-child and couple relationships.

**Gayla Margolin,** PhD, is a professor of Psychology at the University of Southern California. Her research investigates the intergenerational transmission of aggression in young adults as they embark on their own serious romantic relationships. She identifies microprocess behavioral and physiological reactions that can spiral into aggression and that potentially translate into targets for intervention and prevention.

## References

[bibr1-08862605211035877] Archer J. (2000). Sex differences in aggression between heterosexual partners: A meta-analytic review. *Psychological Bulletin*, 126(5), 651–680. https://doi.org/10.1037//0033-2909.126.5.651 10989615 10.1037/0033-2909.126.5.651

[bibr2-08862605211035877] Bartholomew K., & Cobb R. J. (2011). Conceptualizing relationship violence as a dyadic process. In Horowitz L. M. & Strack S. (Eds.), *Handbook of interpersonal psychology: Theory, research, assessment, and therapeutic interventions* (pp. 233–248). John Wiley & Sons Inc.

[bibr3-08862605211035877] Bartholomew K., Cobb R. J., & Dutton D. G. (2015). Established and emerging perspectives on violence in intimate relationships. In Mikulincer M., Shaver P. R., Simpson J. A., & Dovidio J. F. (Eds.), *APA handbook of personality and social psychology, Volume 3: Interpersonal relations* (pp. 605–630). American Psychological Association.

[bibr4-08862605211035877] Bennett D. C., Guran E. L., Ramos M. C., & Margolin G. (2011). College students’ electronic victimization in friendships and dating relationships: Anticipated distress and associations with risky behaviors. *Violence and Victims*, 26(4), 410–429. https://doi.org/10.1891/0886-6708.26.4.410 21882666 10.1891/0886-6708.26.4.410

[bibr5-08862605211035877] Berns S. B., Jacobson N. S., & Gottman J. M. (1999a). Demand–withdraw interaction in couples with a violent husband. *Journal of Consulting and Clinical Psychology*, 67(5), 666–674. https://doi.org/10.1037//0022-006x.67.5.666 10535233 10.1037//0022-006x.67.5.666

[bibr6-08862605211035877] Berns S. B., Jacobson N. S., & Gottman J. M. (1999b). Demand/withdraw interaction patterns between different types of batterers and their spouses. *Journal of Marital and Family Therapy*, 25(3), 337–348. https://doi.org/10.1111/j.1752-0606.1999.tb00252.x 10405919 10.1111/j.1752-0606.1999.tb00252.x

[bibr7-08862605211035877] Berry Mendes W., Gray H. M., Mendoza-Denton R., Major B., & Epel E. S. (2007). Why egalitarianism might be good for your health: Physiological thriving during stressful intergroup encounters. *Psychological Science*, 18(11), 991–998. https://doi.org/10.1111/j.1467-9280.2007.02014.x 17958714 10.1111/j.1467-9280.2007.02014.x PMC2430625

[bibr8-08862605211035877] Bradbury T. N., & Fincham F. D. (1991). A contextual model for advancing the study of marital interaction. In Fletcher G. J. & Fincham F. D. (Eds.), *Cognition in close relationships* (pp. 127–147). Erlbaum.

[bibr9-08862605211035877] Bradley R. P. C., Drummey K., Gottman J. M., & Gottman J. S. (2014). Treating couples who mutually exhibit violence or aggression: Reducing behaviors that show a susceptibility for violence. *Journal of Family Violence*, 29(5), 549–558. https://doi.org/10.1007/s10896-014-9615-4

[bibr10-08862605211035877] Burman B., Margolin G., & John R. S. (1993). America’s angriest home videos: Behavioral contingencies observed in home reenactments of marital conflict. *Journal of Consulting and Clinical Psychology*, 61(1), 28–39. https://doi.org/10.1037//0022-006x.61.1.28 8450105 10.1037//0022-006x.61.1.28

[bibr11-08862605211035877] Caldwell J. E., Swan S. C., & Woodbrown V. D. (2012). Gender differences in intimate partner violence outcomes. *Psychology of Violence*, 2(1), 42–57. https://doi.org/10.1037/a0026296

[bibr12-08862605211035877] Capaldi D. M., & Crosby L. (1997). Observed and reported psychological and physical aggression in young, at-risk couples. *Social Development*, 6(2), 184–206. https://doi.org/10.1111/j.1467-9507.1997.tb00101.x

[bibr13-08862605211035877] Capaldi D. M., Kim H. K., & Shortt J. W. (2007). Observed initiation and reciprocity of physical aggression in young, at-risk couples. *Journal of Family Violence*, 22(2), 101–111. https://doi.org/10.1007/s10896-007-9067-1 17468783 10.1007/s10896-007-9067-1 PMC1858633

[bibr14-08862605211035877] Capaldi D. M., Shortt J. W., & Crosby L. (2003). Physical and psychological aggression in at-risk young couples: Stability and change in young adulthood. *Merrill-Palmer Quarterly*, 49(1), 1–27. https://doi.org/10.1353/mpq.2003.0001

[bibr15-08862605211035877] Cordova J. V., Jacobson N. S., Gottman J. M., Rushe R., & Cox G. (1993). Negative reciprocity and communication in couples with a violent husband. *Journal of Abnormal Psychology*, 102(4), 559–564. https://doi.org/10.1037//0021-843x.102.4.559 8282924 10.1037//0021-843x.102.4.559

[bibr16-08862605211035877] Daff E. S., McEwan T. E., & Luebbers S. (2021). Australian adolescents’ experiences of aggression and abuse by intimate partners. *Journal of Interpersonal Violence*, 36(9-10), NP5586-NP5609. https://doi.org/10.1177/0886260518801936 10.1177/0886260518801936 30261813

[bibr17-08862605211035877] Dardis C. M., Dixon K. J., Edwards K. M., & Turchik J. A. (2015). An examination of the factors related to dating violence perpetration among young men and women and associated theoretical explanations: A review of the literature. *Trauma, Violence, & Abuse*, 16(2), 136–152. https://doi.org/10.1177/1524838013517559 10.1177/1524838013517559 24415138

[bibr18-08862605211035877] Exner-Cortens D., Eckenrode J., & Rothman E. (2013). Longitudinal associations between teen dating violence victimization and adverse health outcomes. *Pediatrics*, 131(1), 71–78. https://doi.org/10.1542/peds.2012-1029 23230075 10.1542/peds.2012-1029 PMC3529947

[bibr19-08862605211035877] Fincham F. D., Garnier P. C., Gano-Phillips S., & Osborne L. N. (1995). Preinteraction expectations, marital satisfaction, and accessibility: A new look at sentiment override. *Journal of Family Psychology*, 9(1), 3-14. https://doi.org/10.1037/0893-3200.9.1.3

[bibr20-08862605211035877] Friend D. J., Bradley R. P. C., & Gottman J. M. (2017). Displayed affective behavior between intimate partner violence types during non-violent conflict discussions. *Journal of Family Violence*, 32(5), 493–504. https://doi.org/10.1007/s10896-016-9870-7

[bibr21-08862605211035877] Garcia R. L., Kenny D. A., & Ledermann T. (2015). Moderation in the actor–partner interdependence model. *Personal Relationships*, 22(1), 8–29. https://doi.org/10.1111/pere.12060

[bibr22-08862605211035877] Gómez A. M. (2011). Testing the cycle of violence hypothesis: Child abuse and adolescent dating violence as predictors of intimate partner violence in young adulthood. *Youth & Society*, 43(1), 171–192. https://doi.org/10.1177/0044118X09358313

[bibr23-08862605211035877] Gordis E. B., Margolin G., & Vickerman K. (2005). Communication and frightening behavior among couples with past and recent histories of physical marital aggression. *American Journal of Community Psychology*, 36(1–2), 177–191. https://doi.org/10.1007/s10464-005-6241-6 16134053 10.1007/s10464-005-6241-6

[bibr24-08862605211035877] Hamberger L. K., Larsen S. E., & Lehrner A. (2017). Coercive control in intimate partner violence. *Aggression and Violent Behavior*, 37, 1–11. https://doi.org/10.1016/j.avb.2017.08.003

[bibr25-08862605211035877] Heyman R. E., & Neidig P. H. (1997). Physical aggression couples treatment. In Halford W. K. & Markman H. (Eds.), *Clinical handbook of marriage and couples’ interventions* (pp. 589–617). Wiley.

[bibr26-08862605211035877] Holtzworth-Munroe A., Stuart G. L., Sandin E., Smutzler N., & Mclaughlin W. (1997). Comparing the social support behaviors of violent and nonviolent husbands during discussions of wife personal problems. *Personal Relationships*, 4(4), 395–412. https://doi.org/10.1111/j.1475-6811.1997.tb00153.x

[bibr27-08862605211035877] Jacobson N. S., Gottman J. M., Waltz J., Rushe R., Babcock J., & Holtzworth-Munroe A. (1994). Affect, verbal content, and psychophysiology in the arguments of couples with a violent husband. *Journal of Consulting and Clinical Psychology*, 62(5), 982–988. https://doi.org/10.1037//0022-006x.62.5.982 7806730 10.1037//0022-006x.62.5.982

[bibr28-08862605211035877] Johnson M. P. (2006). Conflict and control: Gender symmetry and asymmetry in domestic violence. *Violence Against Women*, 12(11), 1003–1018. https://doi.org/10.1177/1077801206293328 17043363 10.1177/1077801206293328

[bibr29-08862605211035877] Johnson W. L., Giordano P. C., Manning W. D., & Longmore M. A. (2015). The age-IPV curve: Changes in intimate partner violence perpetration during adolescence and young adulthood. *Journal of Youth and Adolescence*, 44(3), 708–726. https://doi.org/10.1007/s10964-014-0158-z 25081024 10.1007/s10964-014-0158-z PMC4332391

[bibr30-08862605211035877] Kenny D. A., Kashy D. A., & Cook W. L. (2006). *Dyadic data analysis*. Guilford Press.

[bibr31-08862605211035877] Langhinrichsen-Rohling J., Misra T. A., Selwyn C., & Rohling M. L. (2012). Rates of bidirectional versus unidirectional intimate partner violence across samples, sexual orientations, and race/ethnicities: A comprehensive review. *Partner Abuse*, 3(2), 199–230. https://doi.org/10.1891/1946-6560.3.2.199

[bibr32-08862605211035877] Levenson R. W., & Gottman J. M. (1985). Physiological and affective predictors of change in relationship satisfaction. *Journal of Personality and Social Psychology*, 49(1), 85–94. https://doi.org/10.1037/0893-3200.20.2.256 4020618 10.1037//0022-3514.49.1.85

[bibr33-08862605211035877] Lorber M. F., & O’leary K. D. (2004). Predictors of the persistence of male aggression in early marriage. *Journal of Family Violence*, 19(6), 329–338. https://doi.org/10.1007/s10896-004-0678-5

[bibr34-08862605211035877] Margolin G., John R. S., & Foo L. (1998). Interactive and unique risk factors for husbands’ emotional and physical abuse of their wives. *Journal of Family Violence*, 13(4), 315–344. https://doi.org/10.1023/A:1022880518367

[bibr35-08862605211035877] O'Leary K. D., & Slep A. M. S. (2003). A dyadic longitudinal model of adolescent dating aggression. *Journal of Clinical Child and Adolescent Psychology*, 32(3), 314–327. https://doi.org/10.1207/S15374424JCCP3203_01 12881021 10.1207/S15374424JCCP3203_01

[bibr36-08862605211035877] Paradis A., Hébert M., & Fernet M. (2015). Dyadic dynamics in young couples reporting dating violence: An actor–partner interdependence model. *Journal of Interpersonal Violence*, 32(1), 130–148. https://doi.org/10.1177/0886260515585536 25969443 10.1177/0886260515585536 PMC5149110

[bibr37-08862605211035877] Sanford K. (2006). Communication during marital conflict: When couples alter their appraisal, they change their behavior. *Journal of Family Psychology*, 20(2), 256–265. https://doi.org/10.1037/0893-3200.20.2.256 16756401 10.1037/0893-3200.20.2.256

[bibr38-08862605211035877] Stith S. M., McCollum E. E., Amanor-Boadu Y., & Smith D. (2012). Systemic perspectives on intimate partner violence treatment. *Journal of Marital and Family Therapy*, 38(1), 220–240. https://doi.org/10.1111/j.1752-0606.2011.00245.x 22283388 10.1111/j.1752-0606.2011.00245.x

[bibr39-08862605211035877] Wolfe D. A., Scott K., Reitzel-Jaffe D., Wekerle C., Grasley C., & Straatman A. L. (2001) Development and validation of the Conflict in Adolescent Dating Relationships Inventory. Psychological Assessment, 13(2), 277–293. https://doi.org/10.1037/1040-3590.13.2.277. 11433803

